# PLA2R-positive membranous nephropathy in IgG4-related disease

**DOI:** 10.1186/s12882-024-03511-3

**Published:** 2024-02-23

**Authors:** Yusuke Ushio, Taro Akihisa, Kazunori Karasawa, Momoko Seki, Shizuka Kobayashi, Yoei Miyabe, Hiroshi Kataoka, Naoko Ito, Sekiko Taneda, Shin’ichi Akiyama, Akira Hebisawa, Mitsuhiro Kawano, Kazuho Honda, Junichi Hoshino

**Affiliations:** 1 Department of Nephrology, Tokyo Women’s Medical University, 8-1, Kawada-Cho, Shinjuku-Ku, Tokyo, Japan; 2 Department of Surgical Pathology, Tokyo Women’s Medical University, Tokyo, Japan; 3 Department of Nephrology, Nagoya University Graduate School of Medicine, Nagoya, Japan; 4 Clinical Research Center, National Hospital Organization Tokyo National Hospital, Tokyo, Japan; 5 Department of Rheumatology, Graduate School of Medical Science, Kanazawa University, Kanazawa, Japan; 6 Department of Anatomy, Showa University School of Medicine, Tokyo, Japan

**Keywords:** IgG4-related Disease, PLA2R-positive membranous nephropathy

## Abstract

**Background:**

IgG4-related disease (IgG4-RD) is a fibroinflammatory disease that affects multiple organs, including the pancreas, lacrimal glands, salivary glands, periaortic/retroperitoneum, and kidney. Interstitial nephritis is a typical renal disorder associated with IgG4-RD, but membranous nephropathy is also seen in some cases.

**Case presentation:**

Herein we report on the case of a 77-year-old male patient with nephrotic syndrome and IgG4-related lung disease. His serum phospholipase A2 receptor (PLA2R) antibody was positive. His renal biopsy specimen was also positive for PLA2R.

The renal biopsy specimen showed membranous nephropathy with equal IgG3 and IgG4 immunofluorescence staining and no interstitial nephritis, suggesting IgG4-RD manifesting as membranous nephropathy.

**Conclusions:**

Nephrotic syndrome caused by membranous nephropathy is sometimes associated with IgG4-RD. In such cases, even if serum PLA2R antibody is positive, it should be considered that the membranous nephropathy may be secondary to IgG4-RD.

## Background

The concept of IgG4-related disease (IgG4-RD) was suggested by Kamisawa et al. [1]. It is a systemic disease associated with retroperitoneal fibrosis, sclerosing cholangitis, and autoimmune pancreatitis [2]. Lung lesions were first reported by Duvic et al. in 2004 [3]. IgG4-related lung disease (IgG4-RLD) is clinically classified as either an inflammatory pseudotumor, interstitial pneumonia, organizing pneumonia, or lymphomatous granuloma [4]. Imaging findings are also classified into four categories; however, IgG4-RLD has a variety of clinical and radiological findings and should be differentiated from diseases such as lung cancer, multicentric Castleman’s disease, and interstitial pneumonia [2]. Tubulointerstitial nephritis (TIN) is the most common renal lesion in IgG4-RD, and membranous nephropathy (MN) has been reported in 7% of cases of interstitial nephritis due to IgG4-RD [5].

MN is the most common cause of adult-onset nephrotic syndrome in non-diabetic patients. It frequently develops in older adults and can lead to end-stage kidney disease. It is thought that 70–80% of MN cases are primary MN caused by phospholipase A2 receptor (PLA2R) and thrombospondin type-1 domain-containing 7A (THSD7A). The remaining 20%–30% of cases are thought to be secondary MN, with causes including the hepatitis B virus, systemic lupus erythematosus, sarcoidosis, malignant tumors, and drugs [6].

PLA2R is the major target antigen of podocytes in patients with primary MN, as reported by Beck et al. [7]. Anti-PLA2R antibodies are mainly of the IgG4-subclass and are thought to exist in both blood and glomerular deposits. PLA2R was initially thought to be a specific antigen for primary MN; however, in recent years, there have been sporadic reports of PLA2R antibody-positive cases in secondary MN, and the specificity for primary MN has been reconsidered [8, 9].

We report herein on a case of PLA2R antibody-positive MN without TIN, complicated with IgG4-RD only affecting the lung, as there is a possible relationship between IgG4-RD and PLA2R antibody-positive MN.

## Case presentation

**Fig. 1:**
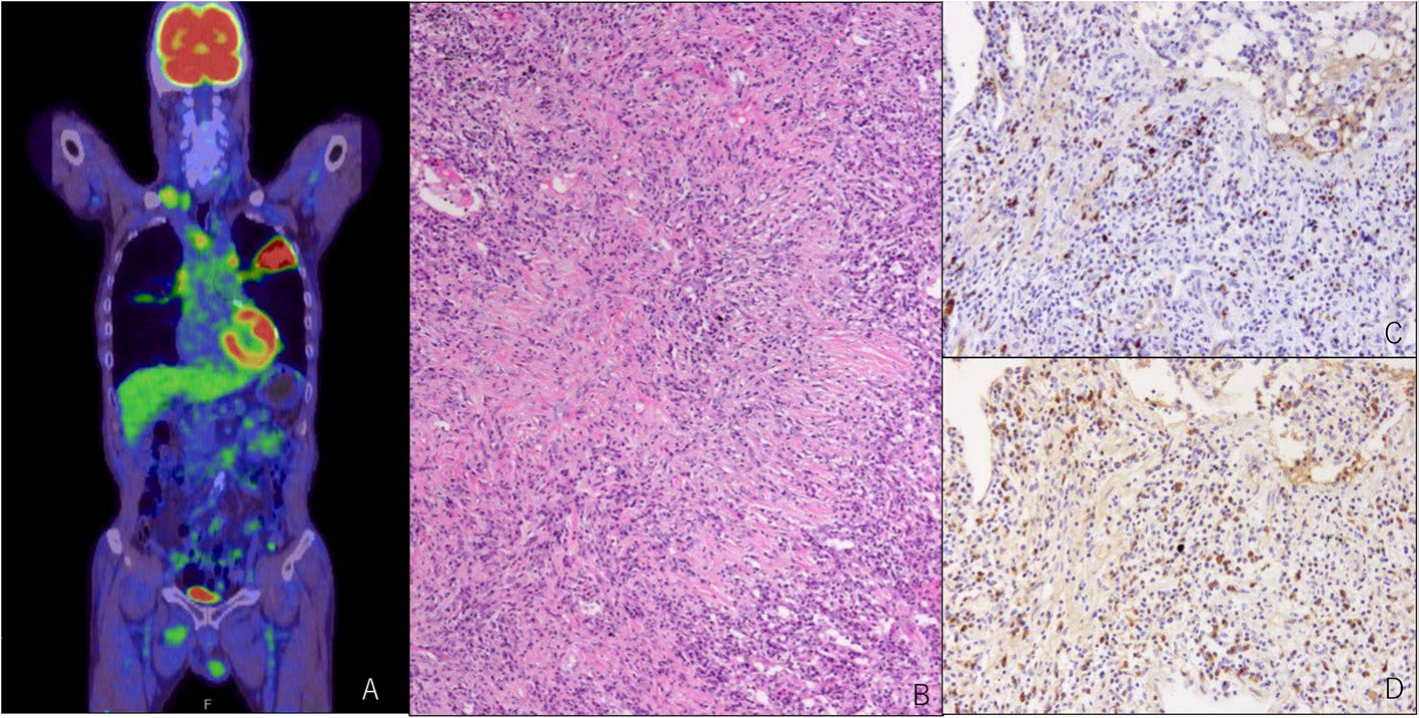
On FDG PET-CT hyperaccumulation was observed in the left lung and right pleura (**A**). Light microscopy of the resected lung tumor shows lymphoplasmacytic proliferation, and storiform fibrosis on intervening stroma. HE staining, original magnification × 100 (**B**). Immunohistochemistry microscopy showed IgG4-positive plasma cells in the specimen,IgG4/IgG ratio 0.81. IgG4 staining, original magnification × 200 (**C**), IgG staining, original magnification × 200 (**D**)

This case involved a 77-year-old male patient with no relevant medical or family history. One year prior to hospitalization, medical examination found an abnormal chest shadow, renal dysfunction, and abnormal urinary findings, and he visited our hospital. A PET-CT scan suggested right lung cancer and multiple lymph node metastases. One month prior to hospitalization, a video-assisted thoracoscopic biopsy of the right upper lobe was conducted. Pulmonary pathological findings showed dense infiltration of inflammatory cells, such as lymphocytes and plasma cells, accompanied by fibrosis. Obliterative phlebitis was observed in some areas. Immunostaining showed a mixture of CD20-positive B-lymphocytes and CD3-positive T-lymphocytes, with no discrepancies between CD4-positive T cells and CD8-positive T cells. IgG4-positive cells were observed, and IgG4/IgG was > 50%, suggesting an IgG4-related disease (Fig. 1). Concurrently, urinary protein was 10.5 g/gCr, uRBC 1–4/HF, NAG 100.1 U/L, urinary β2MG 285 µg/L, serum creatinine (sCr) 1.67 mg/dL, and serum albumin 1.8 g/dL, indicating nephrotic syndrome. The patient was therefore hospitalized.

On hospital admission, the patient had a blood pressure of 140/74 mmHg and no other abnormal vital signs. Pitting edema was observed. Laboratory findings showed a white blood cell count of 7.05 × 10^3^/μL (reference value [RV] 4.0 × 10^3^–8.60 × 10^3^); hemoglobin, 10.4 g/dL (RV 14.0–18.0 g/dL); platelets, 26.1 × 10^4^ /μL (RV 15.0 × 10^4^–35.0 × 10^4^); total protein, 5.2 g/dL (RV 6.5–8.2 g/dL); albumin, 1.8 g/dL (RV 3.8–5.1 g/dL); blood urea nitrogen, 26.8 mg/dL (RV 8.0–20.0 g/dL); sCr, 1.67 mg/dL (RV 0.69–1.06 mg/dL); Na, 143 mEq/L (RV 135–145 mEq/L); K 4.9 mEq/L (3.4–4.9 mEq/L); Cl, 111 mEq/L (98–108 mEq/L); Ca, 7.7 mg/dL (8.5–9.9 mg/dL); and P,3.4 mg/dL (2.5–4.3 mg/dL). IgG was 1154 mg/dL (RV 870–1700 mg/dL); IgG4, 451.3 mg/dL (RV 11–121 mg/dL); serum IgG4/IgG ratio 39.1%; IgA, 175 mg/dL (RV 110–410 mg/dL); IgM, 48 mg/dL (RV 33–190 mg/dL); CH50, 27.2 U/mL (RV 30.0–46.0 U/mL); C3 84.4 mg/dL (RV 65.0–135.0 mg/dL), C4 20.9 mg/dL (RV 13.0–35.0 mg/dL). Antinuclear antibody and anti-double stranded DNA antibody were negative. MPO-ANCA and PR3-ANCA were negative. Serum PLA2R antibodies were 63.1 RU/mL (ELISA, RV < 20 RU/mL). PET-CT scans revealed an infiltrative shadow in the upper lobe of the left lung, as well as mediastinal, bilateral hilar, and right supraclavicular lymphadenopathy, with high uptake observed in all of these.

**Fig. 2:**
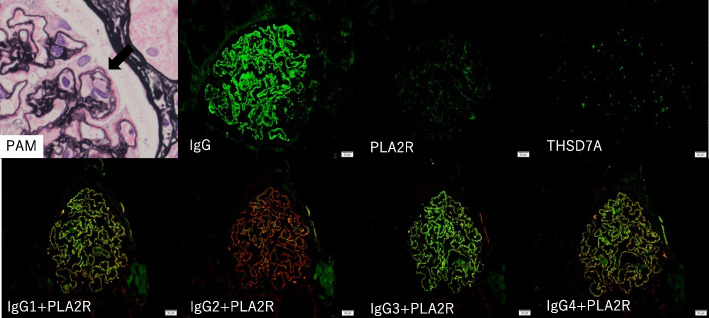
Kidney biopsy findings. Light microscopy showed spike appearances in the glomerular base membrane on periodic acid methenamine-silver staining, × 600. Immunofluorescence microscopy was used for IgG heavy chain, PLA2R, and THSD7A, and double immunofluorescence microscopy for IgG heavy chain subclasses IgG1, IgG2, IgG3, IgG4, and PLA2R, original magnification × 400 (Green: IgG heavy chain subclass, Red: PLA2R)

**Fig. 3:**
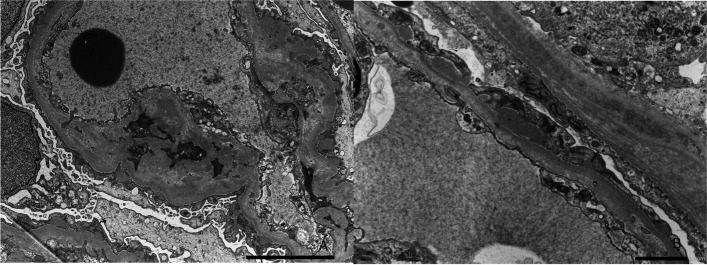
Electron microscopy (EM) showed mesangial electron dense deposits (EDD), subendothelial EDD, increased mesangial matrix, and diffuse foot process effacement, original magnification × 8000 (**A**). EM showed subepithelial EDD and subendothelial edema, original magnification × 12,000(**B**)

A renal biopsy was conducted, and light microscopy revealed 17 glomeruli, three with global sclerosis, thickened and capillary wall double contouring, and spikes and subepithelial deposits in some areas. Inflammatory cell and plasma cell infiltration into the tubulointerstitium were very mild. Immunofluorescence staining showed IgG(2 +), IgA( ±), IgM( +), C1q( ±), C3(2 +), C4( +), and IgG subclasses: IgG1(2 +), IgG2( +), IgG3(2 +), IgG4(2 +), PLA2R( +), THSD7A(-), Nell1(-), SEMA3B(-), κ(2 +), and λ(2 +) (Fig. 2). Electron microscopy (EM) showed mainly subepithelial electron dense deposits (EDD), as well as some intramembranous EDD. Furthermore, EM showed EDD on the mesangium lesion and subendothelial lesion. The pathological diagnosis was MN stage I-II (Fig. 3).

Treatment with prednisolone 30 mg (0.5 mg/kg) was started, and one month later, laboratory data revealed the following: sCr, 1.42 mg/dL; urinary protein, 3.2 g/gCr; IgG/IgG4, 833 mg/dL /207.2 mg/dL; serum PLA2R antibody, 0.7 RU/mL. One year later, after treatment with prednisolone 5 mg, laboratory data revealed the following: sCr, 1.54 mg/dL; urinary protein, 0.15 g/gCr; IgG/IgG4 1131 mg/dL/ 183.6 mg/dL; and a serum IgG4/IgG ratio of 6.1%. The lung lesion disappeared and has not recurred.

## Discussion and conclusions

In MN, 70–80% of cases are primary MN, and antibodies to podocyte antigens PLA2R and THSD7A are indicated in the pathogenesis of primary MN. If PLA2R antibody is positive, then the possibility of primary MN is extremely high. However, PLA2R antibody-positive secondary MN has recently been reported [8, 9]. Primary and secondary MN can be distinguished pathologically by IgG subclass staining and the deposition sites. In IgG subclass staining, IgG1 and IgG4 are dominant in primary MN. However, in secondary MN, due to diseases such as lupus nephritis, IgG1, IgG2, IgG3, and IgG4 are often all stained. Furthermore, subendothelial and mesangial deposition, in addition to subepithelial deposition, suggest a secondary etiology [10]. In this case, immunofluorescence showed dominant staining of IgG3 compared with IgG1, and EM revealed subendothelial edema, as well as findings suggestive of EDD in the mesangium and membrane. Additionally, in IgG subclass staining, co-staining with PLA2R was observed, and PLA2R antibodies were positive; however, it was suggested that this was not a typical primary MN, but MN caused by a secondary factor. In this case, the improvement in urinary protein levels after resolution of the pulmonary lesions also suggest a secondary membranous nephropathy.

IgG4-RD is a fibroinflammatory disease that affects the entire body, commonly including the pancreas, lacrimal glands, salivary glands, kidney, and periaortic/retroperitoneum. In terms of histopathology, plasma cell infiltration and storiform fibrosis are observed regardless of the organ [2]. TIN is the most common renal pathological finding in IgG4-RD, and when progressive renal dysfunction, hypocomplementemia, and characteristic findings in contrast-enhanced CT are observed, then TIN associated with IgG4-RD is suspected. In this case, the patient presented with nephrotic syndrome and pathological findings of membranous nephropathy without TIN. The lung lesions were typical of IgG4-RD and the very high IgG4/IgG levels in the blood were consistent with IgG4-RD.

As previously mentioned, TIN is the most common renal pathologic finding in IgG4-RD, but membranous nephropathy is the most common glomerular lesion, and its relationship to the PLA2R antibody is unknown. As far as we are aware, 13 cases of IgG4-RD with membranous nephropathy without TIN have been reported [11]. It is possible that renal dysfunction at diagnosis is less severe in MN without TIN than in MN with TIN. In cases of MN complicated with TIN, 9/21 (42%) had Cr > 2.0 mg/dL at diagnosis, while 1/13 (7%) had Cr > 2.0 mg/dL in cases of MN without TIN [11]. In addition, three cases of PLA2R antibody-positive MN associated with IgG4-RD have been reported, and serum PLA2R antibody was identified in only one case.

Increased plasma plasmablasts have been suggested in both PLA2R-associated MN [12] and in patients with IgG4-RD [13]. However, the association with the onset of disease is still unclear. Further analysis of the pathogenesis may reveal the relationship between them.

We reported here on a patient with lung-limited IgG4-RD who developed PLA2R antibody-positive membranous nephropathy. Even though the pathology favours primary MN, clinical course of treatment response, is suggestive of MN associated with IgG4-RD. Treatment for IgG4-RD was very effective. But a co-occurence of primary MN with IgG4-RD in the lung which responded to steroid therapy remains a possibility that cannot be completely excluded. Further research is needed to determine the relationship between IgG4-RD and PLA2R antibody positivity.

## Data Availability

Further clinical data and images of this case are available from the corresponding author upon reasonable request.
